# Skin-Conformal Hydrogel-Based Electroencephalography Electrodes with Surfactant-Reorganized PEDOT:PSS

**DOI:** 10.3390/ma18204781

**Published:** 2025-10-19

**Authors:** Ji-Yoon Ahn, Jihyeon Oh, Mi-Ri An, Kun-Woo Nam, Jin-Whan Kim, Sung-Hoon Park

**Affiliations:** Department of Mechanical Engineering, Soongsil University, 369 Sangdo-ro, Dongjak-Gu, Seoul 06978, Republic of Korea; a024679@naver.com (J.-Y.A.); adad55515@soongsil.ac.kr (J.O.); anmiri0622@naver.com (M.-R.A.); kwn1522@naver.com (K.-W.N.); jinwhan5@naver.com (J.-W.K.)

**Keywords:** hydrogel, PEDOT:PSS, TritonX-100, electroencephalography, skin–electrode interface

## Abstract

Electroencephalography (EEG) electrodes require low impedance, high biocompatibility, and long-term performance. Conventional Ag/AgCl wet electrodes achieve low impedance but suffer from dehydration and skin irritation, whereas dry electrodes often induce discomfort or exhibit high impedance. To address these limitations, this study engineered a hydrogel-based electrode by incorporating PEDOT:PSS and the nonionic surfactant Triton X-100 into an acrylic acid hydrogel matrix. The flexible acrylic acid backbone, conductive PEDOT:PSS domains, and the nanofibrillar network promoted by Triton X-100 simultaneously enhanced mechanical compliance and electrical stability. In addition, the structural rearrangement of PEDOT:PSS was verified through morphological analyses. The fabricated electrode exhibited a modulus comparable to human soft tissue, demonstrated strong interfacial adhesion in shear tests, and significantly reduced skin–electrode contact impedance. Furthermore, EEG measurements showed that the hydrogel electrode achieved alpha- and beta-band signal power comparable to commercial Ag/AgCl electrodes. These findings establish the PEDOT:PSS–Triton X-100 hydrogel electrode as a promising candidate to replace conventional wet and dry electrodes for reliable EEG applications.

## 1. Introduction

Electroencephalography (EEG) is a technique that measures and records changes in the brain’s electrical activity from the exterior, primarily reflecting the synaptic potentials of the cerebral cortex [1,2]. EEG signals typically range from a few microvolts to several hundred microvolts [3,4] and are generated by ionic flows during neural transmission [5]. Because these signals provide rich functional information on brain activity, EEG has been widely applied in brain function analysis [6,7,8], clinical diagnostics [9,10,11], cognitive neuroscience [12,13], and bio-signal-based interface technologies [14,15,16]. However, due to their inherently low signal-to-noise ratio (SNR) and high sensitivity to external disturbances, the development of reliable and stable electrode systems remains a critical challenge in EEG research [17,18,19].

Conventional EEG recordings primarily rely on Ag/AgCl-based wet electrodes [20]. While these electrodes achieve low contact impedance through the use of electrolyte gels, they suffer from signal degradation over time as the gels dehydrate, along with issues such as skin irritation and allergic responses [21]. Moreover, the need for repeated gel replenishment, poor wearing comfort, and motion artifacts caused by electrode displacement limit their long-term and daily usability [22,23]. To address these limitations, various dry electrodes have been introduced [24,25,26], yet several challenges remain, such as microneedle electrodes, which provide low impedance but often cause discomfort and skin damage [27], and non-contact electrodes, which exhibit high contact impedance and are vulnerable to environmental noise [28,29]. Therefore, the development of new electrode materials that combine flexibility, biocompatibility, adhesion, and stable signal acquisition is highly desirable.

To overcome these limitations, hydrogel-based electrodes have recently emerged as a promising alternative [30,31]. Hydrogels are composed of hydrophilic polymer chains crosslinked into a three-dimensional network, exhibiting high water content and remarkable mechanical compliance. Such structural characteristics allow the hydrogel to conform intimately to the microscopic irregularities of the skin surface, maintaining stable contact. In addition, the ionically conductive water network within the hydrogel acts as an efficient ionic pathway at the skin–electrode interface, thereby facilitating effective transmission of bioelectrical signals.

In particular, acrylic acid (AA)-based hydrogels exhibit strong adhesion and biocompatibility owing to hydrogen bonding and electrostatic interactions mediated by carboxyl groups (–COOH), making them highly suitable as materials for bioelectrodes. However, hydrogels composed solely of acrylic acid possess inherently low electrical conductivity, which limits their ability to accurately detect subtle potential variations in EEG applications. Therefore, the incorporation of conductive fillers that can enhance electrical performance while retaining the intrinsic flexibility and skin conformity of the hydrogel is essential for developing high-performance, wearable EEG electrodes.

In this context, PEDOT:PSS has attracted considerable attention as a conductive polymer owing to its excellent biocompatibility and water-processability [32,33,34]. However, its intrinsic electrical conductivity is lower than that of other conductive polymers or metal oxides, limiting its ability to achieve high conductivity when used alone. Moreover, when incorporated into hydrogel matrices, PEDOT:PSS often exhibits a trade-off between electrical conductivity and mechanical or adhesive performance. Hence, achieving simultaneous improvement in conductivity, mechanical robustness, and interfacial adhesion remains a critical challenge for PEDOT:PSS-based hydrogels. To enhance conductivity, secondary doping treatments are commonly employed, most notably through the introduction of organic solvents such as ethylene glycol (EG), dimethyl sulfoxide (DMSO) [35,36,37], and methanol [38,39]. Nevertheless, these solvents pose issues of cytotoxicity and skin irritation upon contact, rendering them unsuitable for wearable EEG electrodes. As an alternative, the incorporation of surfactants has been investigated to improve the conductivity of PEDOT:PSS. Among them, the nonionic surfactant Triton X-100, which possesses both hydrophilic and hydrophobic segments, effectively modulates the interaction between PEDOT and PSS. This process disrupts the colloidal structure, promotes the alignment of PEDOT chains, and thereby enhances charge transport pathways [34,40,41].

Recently, various PEDOT:PSS-based hydrogel electrodes have been developed with the aim of enhancing bio signal acquisition performance. For example, a PAA/PEDOT:PSS hydrogel has been reported to exhibit an electrical conductivity of approximately 2 S·m^−1^, a Young’s modulus below 10 kPa, and an adhesion strength of 37 kPa [42]. Moreover, a PPGP hydrogel, synthesized and crosslinked within a PEDOT:PSS matrix using P(N-isopropylacrylamide–(N-(2-hydroxyethyl)acrylamide)–(N-acryloyl-phenylalanine)) (P(NIPAM-HEAA-APA)), showed an electrical conductivity of ~4 S·m^−1^, a Young’s modulus of 4.8 kPa, and an adhesion strength of 93 kPa [43].

In comparison, the Triton X-100-modified PEDOT:PSS hydrogel proposed in this study exhibited an electrical conductivity of 9.1 S·m^−1^, a shear adhesion strength of 22.4 N·cm^−2^, and a Young’s modulus of about 36 kPa, achieving an excellent balance among electrical, mechanical, and adhesive properties. These results indicate that the TX Gel possesses sufficiently well-balanced characteristics for use as a bioelectrode material, surpassing most previously reported PEDOT:PSS-based hydrogels. Unlike conventional solvent doping strategies that raise biocompatibility concerns, this surfactant-based approach offers a safer and more effective route for conductivity enhancement in wearable bioelectrodes. In this study, Triton X-100 was introduced to restructure the internal morphology of PEDOT:PSS and to improve its electrical properties. Therefore, we fabricated hydrogel-based electrodes that simultaneously achieve high conductivity, mechanical flexibility, and skin compatibility.

## 2. Materials and Methods

### 2.1. Materials

To prepare the acrylic acid-based hydrogel, acrylic acid (AA, Korea Chemicals, Seongnam-si, Republic of Korea) was used as the monomer. Ammonium persulfate (APS, Gonggu Science, Seoul, Republic of Korea) and N,N′-methylenebisacrylamide (MBAA, Apex Science, Changzhou, China) were employed as the initiator and crosslinker, respectively. To enhance the electrical performance of the hydrogel, poly(3,4-ethylenedioxythiophene):polystyrene sulfonate (PEDOT:PSS, PH1000, Sigma-Aldrich, Darmstadt, Germany) was incorporated as a conductive filler. The structural rearrangement of PEDOT:PSS was facilitated by introducing the surfactant Triton X-100 (Electron Microscopy Sciences, Hatfield, PA, USA). Deionized water (DI water) was used for all experiments. All reagents were used as received without further purification.

### 2.2. Preparation of Conductive Filler and Fabrication of Hydrogel EEG Electrodes

PEDOT:PSS is widely known as a water-dispersible conductive polymer with excellent biocompatibility. In the colloidal state, the conductive PEDOT chains are surrounded by the PSS matrix acting as a stabilizer, and this structural characteristic limits the electrical conductivity when used as a conductive filler.

**Figure 1:**
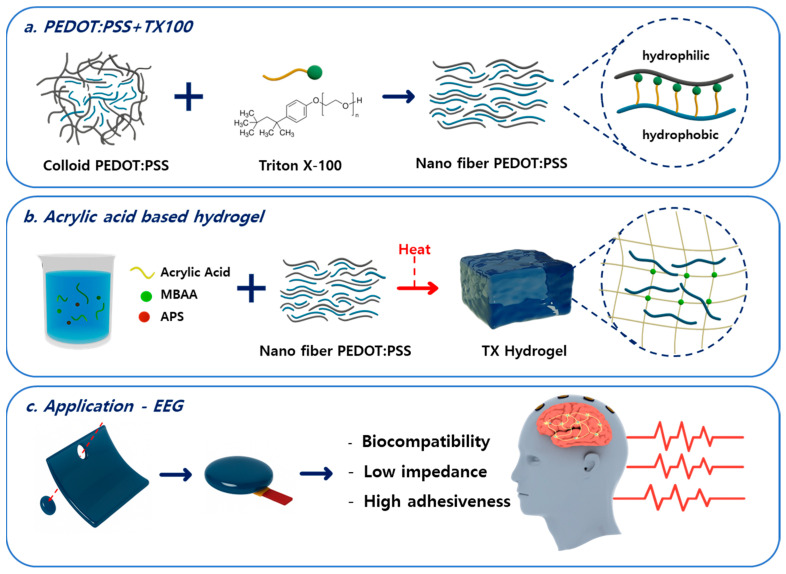
(**a**) Structural rearrangement of PEDOT:PSS induced by the addition of Triton X-100; (**b**) Preparation of acrylic acid-based hydrogel; (**c**) Application of the fabricated hydrogel electrode for electroencephalogram (EEG) recording.

As shown in Figure 1a, the biocompatible nonionic surfactant Triton X-100 was introduced to induce structural rearrangement and uniform dispersion of PEDOT:PSS. Triton X-100 is an amphiphilic molecule possessing both a hydrophilic poly(ethylene oxide) chain and a hydrophobic alkyl phenyl group. When introduced into PEDOT:PSS, it modulates the interactions between the PEDOT and PSS components within the colloidal structure. The hydrophobic tail of Triton X-100 interacts with the hydrophobic π-conjugated chains of PEDOT and penetrates between the PEDOT domains, while the hydrophilic chain stabilizes the system through polar interactions with the PSS regions. During this process, the aggregation of PEDOT particles encapsulated by PSS is partially disrupted, allowing the PEDOT chains to rearrange into a more planar and ordered configuration [44,45]. This structural rearrangement strengthens the π–π stacking interactions within the PEDOT domains and reorganizes them into a nanofibrillar network, thereby extending charge-transport pathways and enhancing the overall electrical performance. The fabrication process of the hydrogel electrode is illustrated in Figure 1b. The hydrogel was synthesized using acrylic acid monomer, ammonium persulfate (APS) as an initiator, and N,N′-methylenebisacrylamide (MBAA) as a crosslinker. First, acrylic acid, APS, and MBAA were mixed to prepare the hydrogel precursor solution. Then, the PEDOT:PSS/Triton X-100 mixture was added and vigorously stirred to achieve uniform dispersion. The mixed solution was poured into a silicone mold and subjected to thermal polymerization at 80 °C. During polymerization, APS generated free radicals to initiate the polymerization of acrylic acid monomers, while MBAA formed crosslinking bonds between polymer chains, resulting in a three-dimensional hydrogel network structure. Numerous pores capable of accommodating water molecules were formed within this structure. The polymerized hydrogel was washed with deionized (DI) water and then swollen in DI water for 24 h. Finally, as shown in Figure 1c, the swollen hydrogel was cut into circular disks with a diameter of 10 mm and used as EEG electrodes. Photographs of the fabricated hydrogels are provided in Supplementary Materials (SM). Figure S1a,b show the hydrogels and Figure S1c presents the hydrogel integrated with the EEG electrode.

### 2.3. Characterization

The characteristics of the hydrogel and the conductive filler PEDOT:PSS were evaluated through morphological, electrical, mechanical, and EEG signal analyses. First, PEDOT:PSS/Triton X-100 mixed solutions were spin-coated onto silicon wafers, and the morphological changes were examined using a field-emission scanning electron microscope (FE-SEM) and an atomic force microscope (AFM). These analyses confirmed the effective transformation of PEDOT:PSS from a colloidal structure into a nanofibrillar network.

The electrical conductivity of the composites was measured using a digital multimeter (DMM7510, Keithley, Solon, OH, USA) in the two-probe mode. To investigate the dynamic electrical response, a customized three-dimensional stretcher (Namil, Incheon, Republic of Korea) was employed, in which the hydrogel samples were gradually elongated from the initial state up to 150% strain while monitoring the resistance changes.

Impedance analysis was conducted in the frequency range of 10–1000 Hz under two conditions: the intrinsic impedance (Z) of the materials and the skin–electrode contact impedance (Zc). Z values were obtained by placing hydrogel specimens (25 × 25 × 2 mm) between copper plates and measuring the resistance across the two surfaces with LCR meter (LCR-6300, GW Instek, New Taipei, Taiwan). Zc was evaluated using hydrogel electrodes fabricated in a circular disk shape (10 mm in diameter), which were attached to the skin. A pair of electrodes was placed 5 cm apart on the forearm, and an alternating current of 5 mV was applied within the frequency range of 10–1000 Hz. For comparison, Zc values of commercial Ag/AgCl electrodes were also measured under identical conditions.

Adhesion strength was assessed by inserting the hydrogel between two copper plates and stretching them with a universal testing machine (UTM) until detachment occurred. The maximum load was recorded, and the tests were repeated ten times. The shear adhesion strength was calculated by dividing the maximum load by the initial bonded area. Characterization was performed in accordance with the ASTM standard test method.

EEG measurements were performed using a QEEG-64FX system (Laxtha, Daejeon, Republic of Korea). Each subject rested for 5 min before measurement, and the electrodes were positioned according to the international 10–20 system. Recording electrodes were placed at the frontal positions Fp1 and Fp2, with A2 used as the reference electrode. Alpha-band signals were analyzed while the subject alternately kept their eyes open for 10 s and closed for 10 s. This open–close sequence was repeated five times per session, and the entire procedure was conducted five independent times for each subject to verify reproducibility and temporal stability. Beta-band signals were analyzed while the subject performed a concentration task (spot-the-difference game). During the beta-wave measurement, each subject performed the concentration task for 5 min followed by a 5 min resting period, and this cycle was also repeated five times. The acquired EEG data were processed using analysis software (TeleScan CD-TS-2.2, LAXTHA, Daejeon, Republic of Korea). All experiments were repeated at least five times for each subject to ensure statistical reliability, and the results are reported as average values.

## 3. Results and Discussion

### 3.1. Morphological Analysis of PEDOT:PSS with Triton X-100

**Figure 2:**
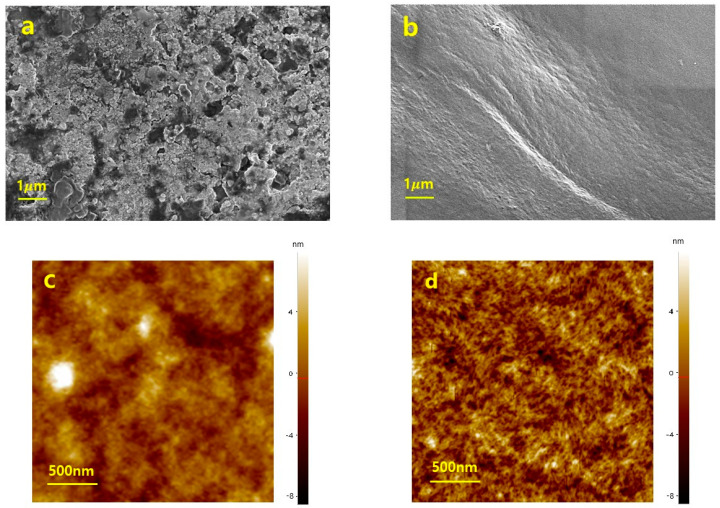
Surface morphology and topographical analysis of PEDOT:PSS films. (**a**) SEM image of pristine PEDOT:PSS; (**b**) SEM image of PEDOT:PSS with Triton X-100; (**c**) AFM image of pristine PEDOT:PSS (**d**) AFM image of PEDOT:PSS with Triton X-100.

PEDOT:PSS inherently exhibits a colloidal structure, which limits the formation of a continuous conductive network. To achieve high conductivity, it is essential for the PEDOT phase to form an interconnected network [46,47]. Thus, breaking up the colloidal domains and inducing structural rearrangement is a critical challenge that must be addressed. As observed in Figure 2a, pristine PEDOT:PSS exhibits an irregular colloidal morphology with aggregated granular domains. The AFM image (Figure 2c) further reveals heterogeneous domains with poorly interconnected aggregates, confirming the limited charge transport pathways in the pristine state. In contrast, Figure 2b demonstrates that PEDOT:PSS with Triton X-100 presents a smoother and denser surface morphology, indicating that structural rearrangement occurred due to the presence of the surfactant. The nanoscale differences are more clearly revealed in the AFM image (Figure 2d), where uniformly distributed nanofibrillar structures are formed and interconnected, thereby establishing an efficient conductive network.

### 3.2. Optimization of Hydrogel Composition and Electrical Properties

**Figure 3:**
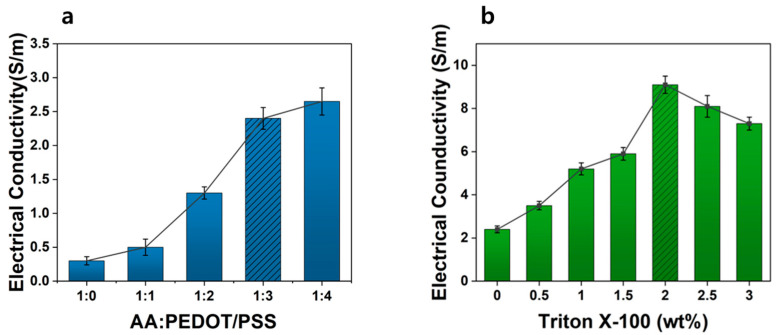
(**a**) Electrical conductivity of hydrogels as a function of PEDOT:PSS contents (*n* = 10); (**b**) Electrical conductivity of hydrogels as a function of TX-100 contents (*n* = 10).

Acrylic acid-based hydrogels inherently exhibit low electrical conductivity, which limits their applicability in biosensor devices. To address this limitation, PEDOT:PSS, a biocompatible conductive filler, was incorporated into an acrylic acid-based hydrogel (AA Gel) to fabricate PEDOT Gel, and the electrical properties were evaluated by varying the weight ratio of PEDOT:PSS to acrylic acid (AA). Figure 3a shows the change in electrical conductivity as a function of the AA:PEDOT:PSS weight ratio. The conductivity of the PEDOT:PSS-free composition (1:0) was as low as 0.3 S/m, but it increased progressively with increasing PEDOT:PSS content. A steep enhancement was observed up to the 1:3 composition, whereas the improvement became limited at 1:4. This behavior can be attributed to the non-uniform dispersion of excess PEDOT:PSS [42,46] within the hydrogel and the detachment of undispersed PEDOT:PSS from the surface during washing.

To further improve the electrical performance, Triton X-100 was introduced into the hydrogel with an AA:PEDOT:PSS ratio of 1:3, producing the TX Gel. Figure 3b presents the electrical conductivity as a function of Triton X-100 content (0–2.5 wt%). Upon the addition of Triton X-100, the colloidal structure of PEDOT:PSS was reorganized into a nanofibrillar morphology, thereby extending charge transport pathways and significantly enhancing conductivity [34,40,41]. The PEDOT Gel without Triton X-100 exhibited a conductivity of 2.4 S/m, whereas the incorporation of 2 wt% Triton X-100 increased the conductivity to 9.1 S/m, representing an approximately 3.8-fold improvement. However, when the Triton X-100 content exceeded 2 wt%, the conductivity decreased, which was attributed to excess nonionic surfactant that failed to interact effectively with PEDOT:PSS and impeded charge transport within the hydrogel matrix. Based on these results, three types of samples were fabricated: AA Gel containing only acrylic acid, PEDOT Gel with an AA:PEDOT:PSS ratio of 1:3, and TX Gel prepared by incorporating 2 wt% Triton X-100 into the PEDOT:PSS network.

### 3.3. Mechanical Characterization of AA, PEDOT, and TX Gels

**Figure 4:**
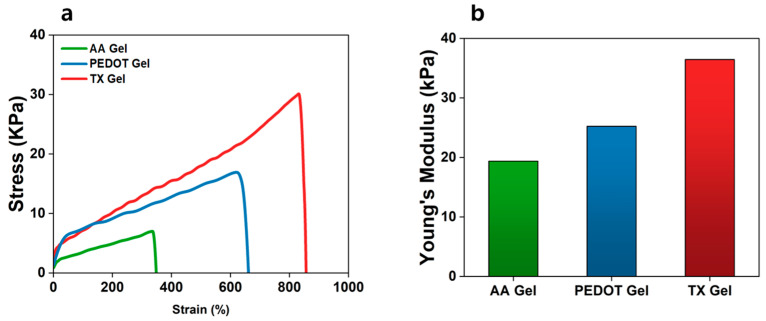
(**a**) Tensile stress–strain curves of AA Gel, PEDOT Gel, and TX Gel (*n* = 10). (**b**) Comparison of Young’s modulus for AA Gel, PEDOT Gel, and TX Gel.

EEG electrodes must remain attached to the skin for extended periods while being subjected to body movements, perspiration, and repeated mechanical deformations such as stretching, compression, and twisting. Insufficient mechanical strength can cause fracture or permanent deformation of the electrode, resulting in unstable skin–electrode contact, increased interfacial impedance, and degraded signal quality. Therefore, achieving both high tensile strength and structural stability is essential for reliable EEG sensors. Figure 4a presents the tensile stress–strain curves of AA Gel, PEDOT Gel, and TX Gel. The interaction between conductive fillers and the acrylic acid framework enhanced both strength and fracture strain. The fracture strength and elongation at break of AA Gel were 7.02 kPa and 361.78%, respectively, whereas PEDOT Gel exhibited increased values of 17.03 kPa and 675%. Notably, TX Gel achieved a fracture strength of 31.2 kPa and an elongation of 856%. These results suggest that the nanofibrillar network formed in the presence of the surfactant facilitates stress transfer while preserving the intrinsic ductility of the hydrogel.

Young’s modulus is another critical parameter directly related to the long-term stability of biocompatible electrodes [48]. As shown in Figure 4b, TX Gel exhibited a modulus of approximately 36 kPa, which lies within the elastic range of human soft tissues (1–100 kPa). Importantly, this mechanical stability was maintained even under high water content, indicating that the hydrogel can preserve functional consistency under repeated deformation. Collectively, these findings indicate that TX Gel possesses mechanical robustness compatible with durable EEG electrode applications.

### 3.4. Evaluating Dynamic Artifacts

**Figure 5:**
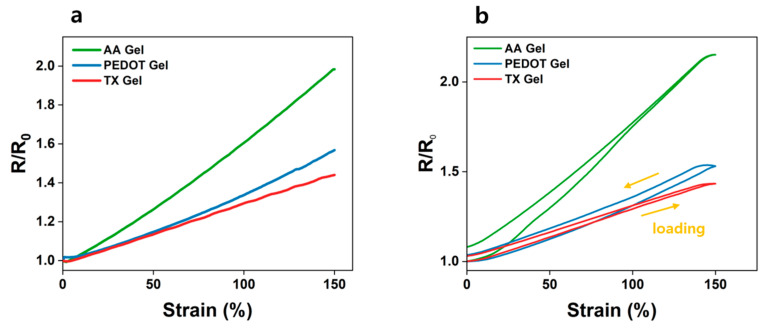
(**a**) Relative resistance change (R/R_0_) of AA Gel, PEDOT Gel, and TX Gel under tensile strain. (**b**) Hysteresis behavior of the hydrogels after 20 cyclic stretching–releasing tests.

Dynamic artifacts can significantly degrade EEG signal quality. To minimize this issue, the hydrogel electrodes were designed to maintain stable conductivity under mechanical deformation. The relative resistance changes (R/R_0_) of AA Gel, PEDOT Gel, and TX Gel were measured under tensile strain up to 150% (Figure 5a). The pristine AA Gel, which lacked conductive fillers, exhibited the highest resistance variation, reaching R/R_0_ = 1.98 at 150% strain, indicating strong sensitivity to deformation. In contrast, PEDOT Gel showed a reduced resistance change of R/R_0_ = 1.57, while TX Gel further decreased the variation to R/R_0_ = 1.44. This improvement is attributed to the uniform dispersion of PEDOT:PSS and the nanofibrillar reorganization induced by Triton X-100, which established more stable conductive pathways under strain.

Figure 5b shows the cyclic loading–unloading response over 20 cycles. The AA Gel displayed the largest hysteresis (ΔR/R_0_ ≈ 1.08), whereas PEDOT Gel and TX Gel exhibited lower values of 1.036 and 1.030, respectively. The minimized hysteresis of TX Gel highlights its superior mechanical–electrical stability under repeated deformation. Collectively, these results indicate that the incorporation of Triton X-100 improves the dispersion and reconfiguration of PEDOT:PSS nanofibrils, effectively buffering tensile stress while preserving conductive networks. As a result, TX Gel maintained nearly constant resistance during dynamic strain, demonstrating its potential as a reliable hydrogel electrode material for EEG applications.

### 3.5. Evaluation of Adhesion Properties

**Figure 6:**
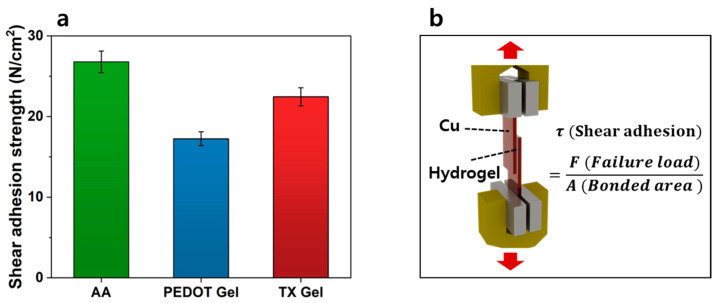
(**a**) Shear adhesion strength of AA Gel, PEDOT Gel, TX Gel (*n* = 10); (**b**) Schematic set-up of the shear adhesion test using a universal testing machine (UTM, DRTECH, Inc., Seongnam-si, Republic of Korea).

The adhesive properties of EEG electrodes are critical for ensuring stable and reliable signal recording. Secure attachment to the skin is particularly important, as insufficient adhesion can lead to electrode displacement, increased skin–electrode impedance, and motion-induced noise. To quantitatively evaluate adhesion performance, shear adhesion strength was measured to simulate the mechanical loads experienced during EEG recording (Figure 6a). The test was conducted by attaching hydrogels between two copper (Cu) substrates and applying load using a universal testing machine (UTM), as illustrated in Figure 6b. All measurements were repeated ten times to obtain average values and error ranges. The measured shear adhesion strengths were 26.78 N/cm^2^ for AA Gel, 17.25 N/cm^2^ for PEDOT Gel, and 22.45 N/cm^2^ for TX Gel. The relatively high adhesion of AA Gel arises from abundant carboxyl groups (–COOH) in acrylic acid, which can readily form hydrogen bonds with amino groups (–NH_2_) in biological tissues [49,50,51]. When PEDOT:PSS was incorporated, adhesion decreased due to the partial coverage of the hydrogel surface by PEDOT:PSS domains, which interfered with hydrogen bond formation. In contrast, the addition of Triton X-100 in TX Gel promoted a more uniform dispersion of PEDOT:PSS within the matrix, reducing surface obstruction and partially recovering adhesion strength. Overall, the adhesion of TX Gel was sufficient to maintain stable attachment to the skin under motion and surface irregularities. This characteristic is particularly advantageous for EEG electrodes that must adhere securely to uneven and hair-covered scalp regions.

### 3.6. Evaluation of Impedance Characteristic

**Figure 7:**
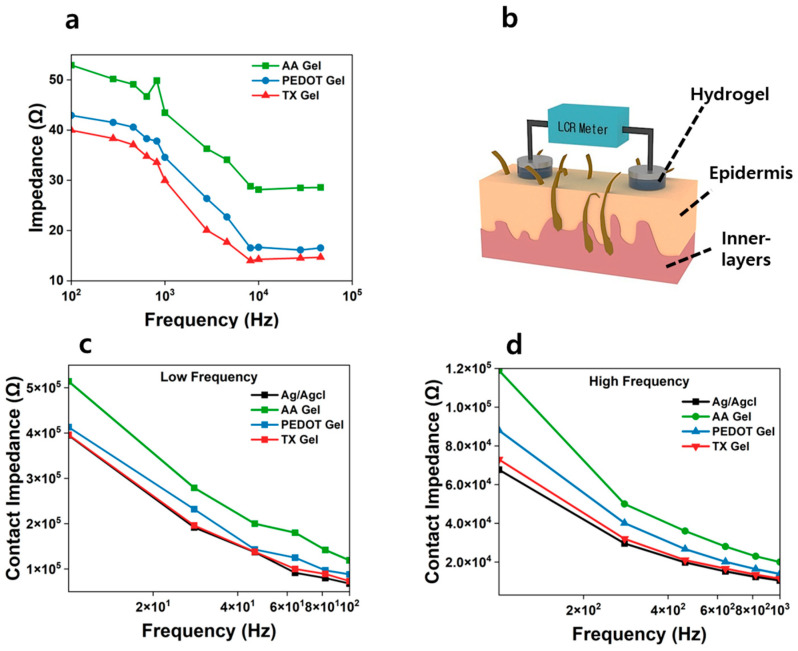
(**a**) Frequency-dependent impedance of AA Gel, PEDOT Gel, and TX Gel electrodes (*n* = 5). (**b**) Schematic illustration of the contact impedance measurement setup using an LCR meter. (**c**) Contact impedance of Ag/AgCl, AA Gel, PEDOT Gel, and TX Gel electrodes in the low-frequency region (*n* = 5). (**d**) Contact impedance of the electrodes in the high-frequency region (*n* = 5).

The skin–electrode contact impedance (Zc) is a critical parameter determining EEG signal quality and is strongly influenced by both the attachment site and the physicochemical properties of the electrode [52,53]. Figure 7a shows the frequency-dependent impedance of AA Gel, PEDOT Gel, and TX Gel. Among them, AA Gel exhibited the highest values, whereas TX Gel consistently showed the lowest impedance due to the formation of continuous conductive pathways.

For comparison with commercial electrodes, Figure 7c,d present the contact impedance of Ag/AgCl, AA Gel, PEDOT Gel, and TX Gel in both the low-frequency (<100 Hz) and high-frequency (100–1000 Hz) ranges. Across all frequencies, TX Gel exhibited impedance values closest to those of Ag/AgCl, whereas AA Gel showed the poorest performance. These results confirm that the nanofibrillar conductive network induced by Triton X-100 not only enhances electrical conductivity but also improves skin conformability, thereby effectively expanding the contact area and reducing Zc. In addition, the superior flexibility of TX Gel allowed it to dissipate stress and accommodate skin motion, which minimized motion-induced artifacts. Collectively, these findings highlight that TX Gel electrodes provide low-impedance and stable signal acquisition comparable to commercial wet electrodes, underscoring their strong potential as a next-generation EEG electrode material.

### 3.7. Recording EEG Signals

**Figure 8:**
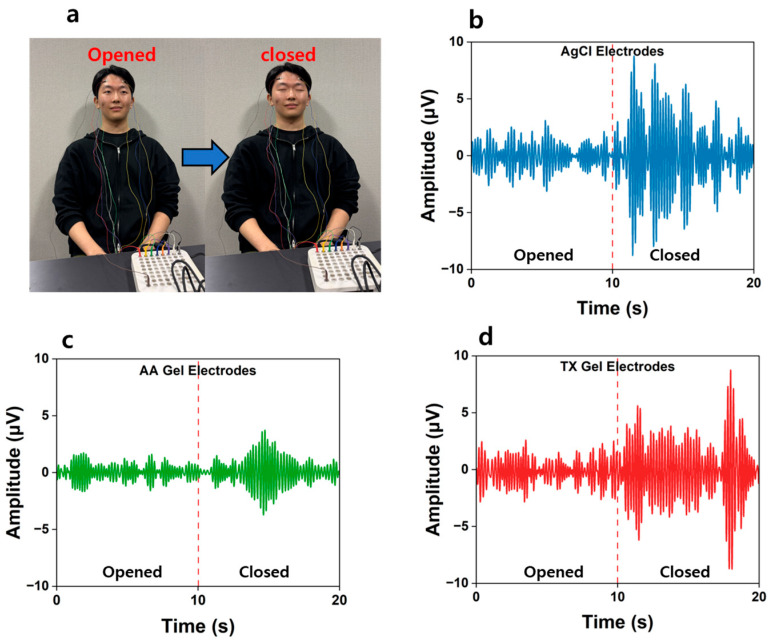
(**a**) Experimental setup of the subject with electrodes attached, EEG alpha wave signals recorded (*n* = 5) during eyes opened and closed using (**b**) AgCl electrodes. (**c**) AA Gel electrodes (**d**) TX Gel electrodes.

**Figure 9:**
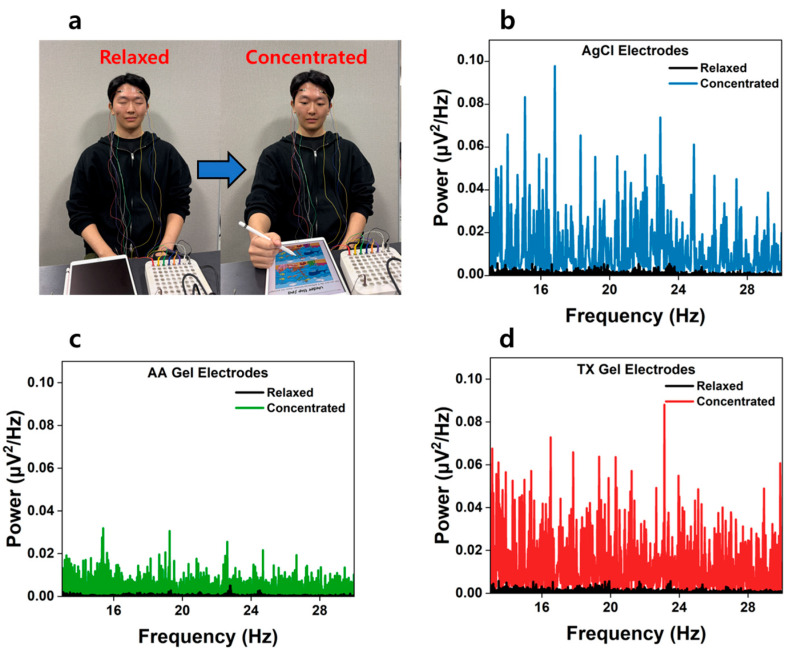
(**a**) Experimental setup showing the subject performing tasks under relaxed and concentrated conditions, EEG beta wave signals recorded (*n* = 5) during relaxed and concentrated using (**b**) AgCl electrodes. (**c**) AA Gel electrodes; (**d**) TX Gel electrodes.

EEG signals were analyzed using an FFT-based power spectrum approach, with alpha and beta bands defined as 8–12 Hz and 13–30 Hz, respectively. Two standard paradigms were employed: (i) eyes-open/eyes-closed for alpha, and (ii) resting/goal-directed task (spot-the-difference) for beta (Figure 8 and Figure 9).

To evaluate the EEG signal acquisition performance of the developed hydrogel electrodes, participants were instructed to alternately open and close their eyes, as shown in Figure 8a. When the eyes were closed, alpha activity was significantly enhanced compared with the eyes-open state. Since alpha rhythms are generally associated with relaxed brain states, these results confirm that the developed electrodes can reliably capture physiologically relevant alpha activity. As shown in Figure 8, the signal amplitude obtained with the AA Gel electrodes was smaller than that of commercial Ag/AgCl electrodes, whereas the TX Gel electrodes exhibited amplitudes comparable to those of the Ag/AgCl electrodes. Furthermore, the enhancement of alpha-band activity upon eye closure was clearly observed with the TX Gel electrodes, reaching a level similar to that of commercial electrodes.

In addition, beta-band activity was analyzed under both resting and task-performing conditions, as illustrated in Figure 9a. Figure 9b–d compare the power spectral densities of beta waves during rest and concentration (spot-the-difference task). A distinct increase in beta power was observed when participants engaged in the concentration task. Notably, the TX Gel electrodes exhibited beta-band power and amplification ratios comparable to those recorded by Ag/AgCl electrodes, thereby demonstrating their ability to detect task-related EEG responses with high fidelity.

The quantitative analysis results are summarized in Table 1 and Table 2. The relative ratio of the closed-eye to open-eye condition increased by approximately 2.5-fold for Ag/AgCl electrodes, 1.58-fold for AA Gel electrodes, and 2.31-fold for TX Gel electrodes (Table 1). This indicates that the TX Gel electrodes achieved alpha wave amplification comparable to that of commercial Ag/AgCl electrodes.

Furthermore, a similar trend was observed in the power spectral density (PSD) analysis of beta-band activity. Under concentration conditions, beta power increased by approximately 22.5-fold for Ag/AgCl electrodes, 15.7-fold for AA Gel electrodes, and 22.2-fold for TX Gel electrodes (Table 2). Notably, the amplification ratio of the TX Gel electrodes was nearly identical to that of the Ag/AgCl electrodes, confirming their ability to effectively capture beta-band signals during cognitive tasks.

These findings strongly support that the TX Gel electrodes, while maintaining inherent advantages of flexibility and biocompatibility, provide EEG signal quality and sensitivity comparable to those of commercial Ag/AgCl electrodes, suggesting their potential as a promising alternative for practical EEG applications.

## 4. Conclusions

In this study, we developed hydrogel-based electrodes by incorporating PEDOT:PSS and the surfactant Triton X-100 into an acrylic acid matrix, which is specifically tailored for electroencephalography applications. The surfactant-driven reorganization of PEDOT:PSS established a nanofibrillar conductive network, which enabled the simultaneous realization of high conductivity, mechanical compliance, and strong interfacial adhesion—a combination rarely realized in conventional electrode designs. The fabricated electrodes exhibited low skin–electrode contact impedance and effective suppression of motion artifacts, ensuring stable recordings even under dynamic conditions. They also captured alpha- and beta-band signals with fidelity comparable to commercial Ag/AgCl electrodes, confirming the practical reliability of this approach. Moreover, the combination of skin conformability, electrical stability, and long-term usability highlights the distinct advantage of this surfactant-based strategy over conventional solvent-doping methods. Overall, this work demonstrates that PEDOT:PSS–Triton X-100 hydrogel electrodes provide a bioelectrode platform that bridges the gap between wet and dry electrodes and offers a promising route for next-generation wearable and implantable bioelectronic interfaces.

## Figures and Tables

**Table 1:** Comparison of mean absolute EEG amplitudes of Alpha wave and relative ratios for different electrode types under eyes-open and eyes-closed conditions.

Electrode Type	Condition	Mean Absolute Amplitude (μV)	Relative Ratio
Ag/AgCl	Eyes Opened	0.8598	1
	Eyes Closed	2.1501	2.5
AA Gel	Eyes Opened	0.2542	1
	Eyes Closed	0.4011	1.58
TX Gel	Eyes Opened	0.8634	1
	Eyes Closed	1.9958	2.31

**Table 2:** Comparison of mean EEG power of Beta wave and relative ratios across electrode types during resting and concentration states.

Electrode Type	Condition	Mean Power (μV^2^/Hz)	Relative Ratio
Ag/AgCl	Resting	0.000527556	1
	Concentration	0.011846486	22.5
AA Gel	Resting	0.000216084	1
	Concentration	0.003402649	15.7
TX Gel	Resting	0.000525723	1
	Concentration	0.011672563	22.2

## Data Availability

The original contributions presented in this study are included in the article/Supplementary Materials. Further inquiries can be directed to the corresponding author.

## Supplementary Materials

The following supporting information can be downloaded at: https://www.mdpi.com/article/10.3390/ma18204781/s1, Figure S1: (a) Photographs of the acrylic acid–based hydrogel (AA Gel), the PEDOT:PSS–Triton X-100 composite hydrogel (TX Gel), and the TX Gel cut to the electrode dimensions for EEG measurement, (b) Side-view images of the same samples showing thickness and uniformity, (c) Photograph showing the TX Gel directly attached to the EEG electrode surface for EEG recording.
