# Supplementary material for: Skin-Conformal Hydrogel-Based Electroencephalography Electrodes with Surfactant-Reorganized PEDOT:PSS
Source: Materials (Basel). 2025 Oct 19;18(20):4781. doi: 10.3390/ma18204781 (PMC12566280; doi:10.3390/ma18204781)
Supplement: Supplementary file 1 [file materials-18-04781-s001.zip › materials-3910858-supplementary.pdf]

Supplementary Information

Skin-Conformal Hydrogel-Based Electroencephalography Electrodes with Surfactant-Reorganized PEDOT:PSS

Ji-Yoon Ahn, Jihyeon Oh, Mi-Ri An, Kun-Woo Nam, Jin-Whan Kim and Sung-Hoon Park *

Department of Mechanical Engineering, Soongsil University, 369 Sangdo-ro, Dongjak-Gu, Seoul 06978, Republic of Korea; a024679@naver.com (J.-Y.A.); adad55515@soongsil.ac.kr (J.O.);
anmiri0622@naver.com (M.-R.A.); kwn1522@naver.com (K.-W.N.); jinwhan5@naver.com (J.-W.K.)
* Correspondence: leopark@ssu.ac.kr; Tel.: +82-2-828-7021

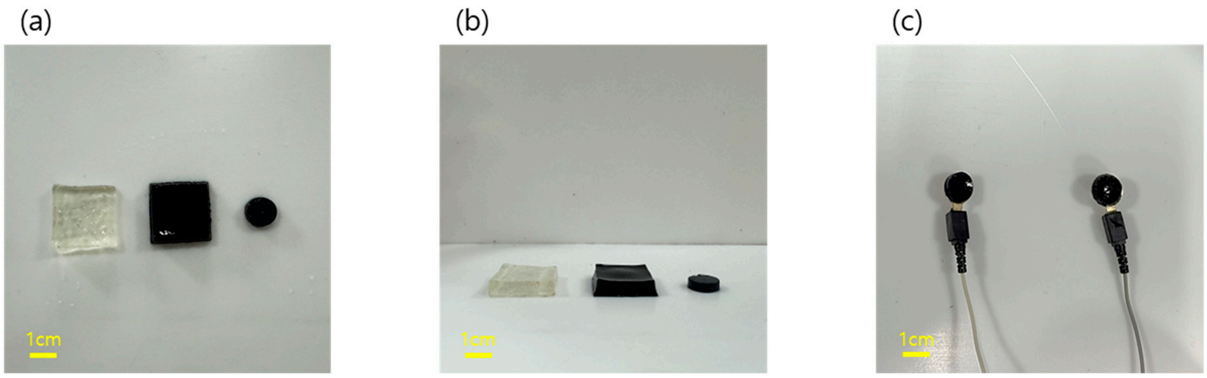

Figure S1. (a) Photographs of the acrylic acid–based hydrogel (AA Gel), the PEDOT:PSS–Triton X-100 composite hydrogel (TX Gel), and the TX Gel cut to the electrode dimensions for EEG measurement, (b) Side-view images of the same samples showing thickness and uniformity, (c) Photograph showing the TX Gel directly attached to the EEG electrode surface for EEG recording.
